# A Clinical and Laboratory Comparison of Varicella-Zoster Virus (VZV) and Epstein-Barr Virus (EBV): Two Cases of Herpesvirus Meningitis in Immunocompetent Adults and a Literature Review

**DOI:** 10.7759/cureus.114529

**Published:** 2026-08-14

**Authors:** Israa A Serhal, Sara Najib, Ibrahim Serhal, Ghenwa K El Dakdoukii

**Affiliations:** 1 Internal Medicine, Hammoud Hospital University Medical Center, Sayda, LBN; 2 internal Medicine, Beirut Arab University, Beirut, LBN; 3 Internal Medicine, Lebanese American University, Beirut, LBN; 4 Infectious Disease, Beirut Arab University, Beirut, LBN

**Keywords:** aseptic meningitis, epstein-barr virus (ebv), immunocompetent adults, lymphocytic meningitis, varicella-zoster virus (vzv)

## Abstract

Varicella-zoster virus (VZV) and Epstein-Barr virus (EBV) are uncommon causes of aseptic meningitis in immunocompetent individuals. We report two cases of polymerase chain reaction (PCR)-confirmed viral meningitis caused by VZV and EBV in previously healthy young adults and compare their clinical presentation, cerebrospinal fluid (CSF) findings, neuroimaging, treatment response, and outcomes with previously reported cases. Both patients presented with lymphocytic meningitis and normal neuroimaging findings. The patient with VZV meningitis experienced rapid clinical improvement following acyclovir therapy and developed a vesicular rash two days after symptom onset, whereas the patient with EBV meningitis presented with a broader range of systemic and neurological symptoms and showed a more gradual recovery. Despite a shared lymphocytic CSF pattern, the two cases demonstrated distinct clinical and laboratory profiles. These findings highlight the importance of molecular testing for definitive diagnosis and support consideration of both viruses in the differential diagnosis of aseptic meningitis in immunocompetent patients.

## Introduction

Viral meningitis is a common cause of aseptic meningitis and is most frequently associated with enteroviruses and members of the Herpesviridae family [1,2]. Among herpesviruses, varicella-zoster virus (VZV) and Epstein-Barr virus (EBV) are recognized but relatively uncommon causes of central nervous system (CNS) infection. Both viruses are neurotropic DNA viruses capable of establishing lifelong latency following primary infection and may reactivate later in life, resulting in a broad spectrum of neurological manifestations ranging from isolated meningitis to encephalitis, vasculopathy, myelitis, and cranial neuropathies [2].

VZV is an enveloped, double-stranded DNA virus belonging to the Herpesviridae family, characterized by a linear genome enclosed within an icosahedral capsid. Following primary infection, which manifests as varicella (chickenpox), VZV establishes lifelong latency within sensory and autonomic ganglia, including the cranial nerve and dorsal root ganglia. Viral reactivation may occur when VZV-specific cell-mediated immunity wanes, particularly with advancing age or immunosuppression. This reactivation results in herpes zoster (shingles), a condition typically characterized by neuropathic pain and a unilateral vesicular eruption confined to one or more dermatomes [3-5]. Although varicella is generally a self-limiting disease of childhood, VZV infection may lead to serious complications, including CNS involvement, pneumonia, and secondary bacterial infections. Disease severity is increased among older adults, pregnant women, and immunocompromised individuals; however, severe morbidity and mortality may also occur in otherwise immunocompetent hosts [4,5].

EBV is a double-stranded DNA virus of the Gammaherpesvirus family with a genome of approximately 172 kb. The viral particle is composed of a DNA-containing core enclosed within an icosahedral nucleocapsid, a protein-rich tegument layer, and an outer lipid envelope that contains virus-encoded glycoproteins. EBV exists as two major types, EBV-1 and EBV-2, which differ primarily in the sequences of genes encoding several Epstein-Barr nuclear antigens, including EBNA-2, EBNA-3A, EBNA-3B, and EBNA-3C. The virus predominantly infects B lymphocytes and nasopharyngeal epithelial cells in immunocompetent hosts [6]. Following infection, EBV can establish either latent infection or undergo lytic replication, each characterized by distinct patterns of viral gene expression. During latency, the virus expresses several non-coding RNAs, including EBV-encoded RNAs (EBER1 and EBER2), whereas lytic infection is associated with the expression of genes such as BART and BHRF1, which produce microRNAs that contribute to viral persistence, host-cell regulation, and the pathogenesis of EBV infection [6].

The aim of this report is to compare the clinical presentation, laboratory profile, cerebrospinal fluid (CSF) findings, treatment response, and short-term outcomes of VZV and EBV meningitis in two immunocompetent young adults, and to contextualize these findings through comparison with previously reported cases.

To our knowledge, direct clinical and CSF comparison between EBV meningitis and VZV meningitis in immunocompetent adults remains limited, as most published data focus on isolated cases, pathogen detection studies, or broader herpesvirus CNS infection cohorts [7-26].

## Case presentation

Case 1

A 34-year-old previously healthy male smoker presented with a three-day history of severe frontal headache. The headache was sudden in onset, persistent, non-radiating, and progressively worsened to a severity of 8/10. It was unresponsive to paracetamol and was associated with blurred vision, one episode of non-projectile vomiting, lethargy, and chills. The patient denied photophobia, phonophobia, altered mental status, dizziness, neck pain or stiffness, abnormal movements, focal neurological deficits, skin rash, weight changes, or other constitutional symptoms. He also denied gastrointestinal, respiratory, or urinary symptoms. There was no history of recent trauma, travel, medication overuse, chickenpox, or herpes zoster.

On admission, the patient had a documented fever of 38.5°C. The remainder of his vital signs were within normal limits. Neurological examination was unremarkable, with no focal deficits and negative meningeal signs. Skin examination revealed no rash or other cutaneous lesions. Cardiovascular, respiratory, and abdominal examinations were normal.

Initial laboratory investigations revealed a white blood cell count of 9,000/μL, with neutrophilic predominance (87.8%), lymphocytes of 7.6%, C-reactive protein (CRP) of 6.5 mg/L, erythrocyte sedimentation rate (ESR) of 20 mm/h, aspartate aminotransferase (AST) of 85 U/L, and alanine aminotransferase (ALT) of 52 U/L.

A non-contrast computed tomography (CT) scan of the brain showed no acute intracranial abnormalities. Lumbar puncture was performed to evaluate for CNS infection. CSF analysis revealed turbid fluid, with a white blood cell count of 380 cells/μL, 99% lymphocytes, no red blood cells, a glucose level of 69 mg/dL, serum glucose level of 100 mg/dL (>50% of the corresponding serum glucose concentration), and protein concentration of 160 mg/dL, consistent with lymphocytic meningitis.

CSF bacterial cultures and a multiplex viral polymerase chain reaction (PCR) panel were obtained, and intravenous acyclovir therapy was initiated empirically. CSF bacterial cultures remained negative, while CSF PCR detected VZV. Blood cultures were also negative.

**Figure 1:**
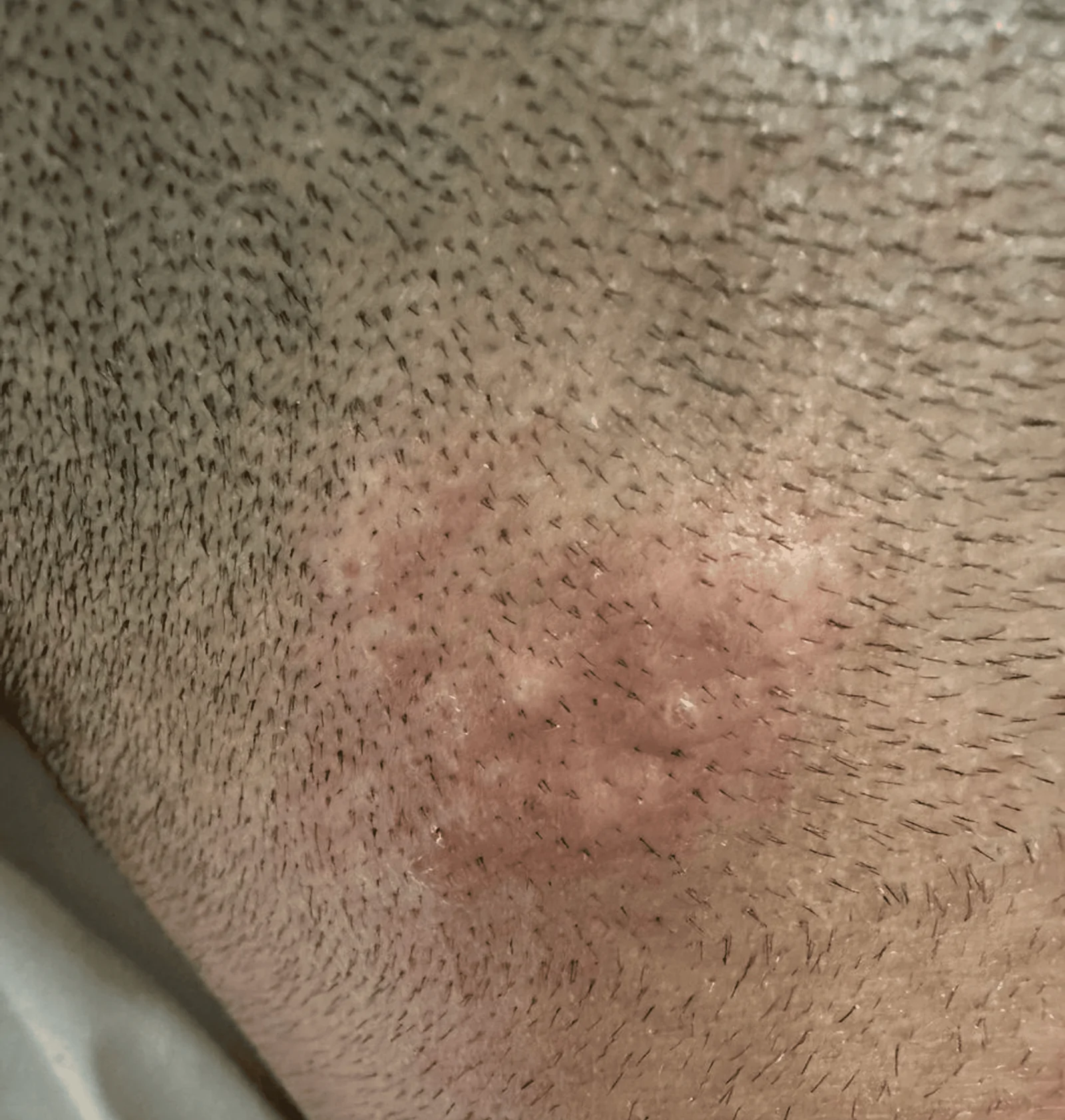
Delayed appearance of occipital herpes zoster rash in a patient with PCR-confirmed VZV meningitis VZV: Varicella-zoster virus

Two days after admission, the patient developed a localized erythematous vesicular rash over the right occipital scalp region (Figure 1). The lesions consisted of clustered vesicles on an erythematous base and remained confined to the occipital area without evidence of dissemination. This delayed appearance of the rash following the onset of neurological symptoms supported the diagnosis of VZV meningitis.

The patient's headache resolved by the third day of hospitalization, accompanied by complete resolution of fever and chills. Inflammatory markers began to decrease on the second day of treatment. He completed a 10-day course of intravenous acyclovir and was discharged without neurological sequelae.

Case 2

A 20-year-old previously healthy non-smoking male presented with a three-day history of headache associated with fever, dizziness, nausea, vomiting, diarrhea, and sore throat. He denied photophobia, neck stiffness, altered mental status, seizures, focal neurological deficits, respiratory symptoms, urinary symptoms, recent travel, or sick contacts.

Physical examination revealed vertical nystagmus and gait instability. Motor strength, sensory examination, cranial nerve function, and cerebellar testing were otherwise normal. Meningeal signs were absent. Oropharyngeal examination showed bilateral tonsillar exudates. No cervical lymphadenopathy or hepatosplenomegaly was detected. Vital signs were within normal limits.

Laboratory investigations demonstrated a white blood cell count of 10,000/μL with 42% neutrophils and 46% lymphocytes. CRP was 4.2 mg/L, with ESR of 25 mm/h, AST of 80 U/L, ALT of 151 U/L, alkaline phosphatase of 83 U/L, gamma-glutamyl transferase (GGT) of 78 U/L, total bilirubin of 0.9 mg/dL, and direct bilirubin of 0.3 mg/dL.

Lumbar puncture was performed because of suspected central nervous system infection. CSF analysis revealed clear, colorless fluid, with a white blood cell count of 70 cells/μL, 97% lymphocytes, no red blood cells, glucose level of 55 mg/dL, serum glucose level of 94 mg/dL (>50% of the corresponding serum glucose concentration), and protein concentration of 213 mg/dL, consistent with lymphocytic meningitis.

Brain CT and magnetic resonance imaging (MRI) with gadolinium contrast showed no acute intracranial abnormalities and no radiological evidence of encephalitis.

Empirical treatment with intravenous acyclovir, ceftriaxone, and vancomycin was initiated. CSF bacterial cultures remained negative. CSF PCR detected EBV DNA at a low level, while serum testing was positive for EBV IgM and IgG antibodies. Cytomegalovirus (CMV) IgM and IgG antibodies were negative. Brucella serology, stool studies, stool cultures, and throat bacterial cultures were all negative.

Gastrointestinal symptoms resolved within the first day of hospitalization, while dizziness and gait instability gradually improved by the fifth hospital day. Inflammatory markers began to decrease by day seven. The patient completed a 10-day course of intravenous acyclovir, while ceftriaxone and vancomycin were discontinued after five days following exclusion of bacterial meningitis. He recovered fully without neurological complications.

To highlight the similarities and differences between VZV and EBV meningitis, the key clinical and cerebrospinal fluid findings of both cases are summarized in Tables 1-2.

**Table 1:** Comparative clinical characteristics of patients with VZV and EBV meningitis CSF: Cerebrospinal fluid; EBV: Epstein-Barr virus; PCR: Polymerase chain reaction; VZV: Varicella-zoster virus

Variable	VZV meningitis	EBV meningitis
Age/Sex	34/M	20/M
Headche	Yes	Yes
Fever	Severe	Moderate
Rash	Yes (appeared after 2 days)	No
Sore throat	No	Yes
Neurological findings	None	Vertigo, gait instability, vertical nystagmus
Peripheral blood	Lymphopenia	Lymphocytosis
Liver function tests	Mildly elevated	Elevated
CT/MRI	Normal	Normal
CSF PCR	VZV-positive	EBV-positive
Treatment	IV acyclovir	IV acyclovir
Clinical improvement	Day 3	Days 5-7
Outcome	Complete recovery	Complete recovery

**Table 2:** Comparative cerebrospinal fluid findings

Variable	Reference range	VZV meningitis	EBV meningitis
CSF WBC (cells/mL)	0-5	380	70
CSF Lymphocytes (%)	-	99	97
CSF Protein (mg/dL)	15-45	160	213
CSF Glucose/serum glucose ratio	≥0.6 (typically 0.6-0.7)	0.69	0.58

## Discussion

CNS disorders are among the major causes of mortality worldwide. Meningitis contributes to approximately 0.6% of global deaths, while survivors often face persistent neurological disabilities. Although several risk factors have been implicated in the pathogenesis of CNS diseases, the impact of viral infections has emerged as an area of significant clinical and research interest [1,2,7].

Meningitis is an inflammatory disease of the meninges that may be classified as bacterial or aseptic. Aseptic meningitis is most commonly viral, although fungal infections, drug-induced inflammation, autoimmune disease, and other non-bacterial causes may also be implicated. Clinically, meningitis often presents with fever, headache, and nuchal rigidity, and bacterial causes must be recognized promptly because delayed antimicrobial therapy may lead to serious systemic complications and long-term neurological sequelae [26,27]. In recent decades, routine childhood vaccination and chemoprophylaxis strategies have reduced the incidence of several forms of bacterial meningitis, making viral meningitis a relatively frequent cause of aseptic meningitis in adults [17,18]. Viral meningitis is typically characterized by cerebrospinal fluid pleocytosis with negative routine bacterial cultures, and enteroviruses remain the most common cause; however, herpesviruses, including VZV and EBV, are important etiologies because they may require targeted molecular diagnosis and specific therapeutic decisions [17,18]. Early lumbar puncture, microbiological testing, and cerebrospinal fluid PCR testing help identify the causative virus and may support rational de-escalation of unnecessary antibacterial or antiviral therapy when interpreted in the appropriate clinical context [18,19].

The present report compares two uncommon causes of aseptic meningitis, VZV and EBV, in immunocompetent adults. Although both patients presented with lymphocytic meningitis and favorable outcomes, notable differences were observed in clinical presentation, CSF findings, and rate of recovery.

VZV, a neurotropic human alphaherpesvirus, is the causative agent of varicella and herpes zoster and represents an important cause of CNS infection. VZV-associated neurological disease includes meningitis, encephalitis, vasculopathy, cerebellitis, myelopathy, and cranial neuropathies, and may occur with or without the characteristic vesicular rash [3-5]. Although VZV meningitis is relatively uncommon, it is increasingly recognized as a cause of aseptic meningitis in both immunocompromised and immunocompetent individuals, including adolescents, young adults, and older adults. PCR-based studies suggest that VZV may account for a meaningful proportion of aseptic meningitis cases, supporting the role of cerebrospinal fluid PCR testing for definitive diagnosis [7-9]. Clinically, VZV meningitis may be difficult to suspect at presentation because the rash may be absent, subtle, or delayed; this is consistent with our patient, who developed a localized occipital vesicular rash only after the onset of neurological symptoms [8-12,20,21]. CSF findings usually show lymphocytic pleocytosis with elevated protein and preserved glucose, although hypoglycorrhachia has also been reported, indicating that CSF glucose alone cannot reliably distinguish VZV meningitis from other infectious etiologies [9,22]. Recent adult studies suggest that isolated VZV meningitis generally has a favorable prognosis, particularly when distinguished from VZV meningoencephalitis, vasculopathy, or other complicated CNS manifestations [4,5,19].

Our VZV case shared several similarities with the case series reported by Chen et al., in which four immunocompetent adults presented with VZV meningitis. Similar to our patient, one individual developed the characteristic vesicular rash only after the onset of neurological symptoms, with the rash appearing two days after the initial presentation of meningitis. This observation highlights that VZV meningitis may precede the appearance of cutaneous manifestations, potentially delaying clinical suspicion and diagnosis. Furthermore, the majority of patients in that series (75%) had no cutaneous lesions at all, emphasizing that the absence of rash does not exclude VZV infection of the CNS [8].

EBV, a member of the Herpesviridae family, infects most individuals worldwide and is frequently asymptomatic; however, symptomatic disease is more commonly observed in children, adolescents, and immunocompromised patients [6]. Although EBV-associated CNS disease is uncommon, it has a broad neurological spectrum that includes aseptic meningitis, encephalitis, cerebellitis, cranial neuropathies, myelitis, Guillain-Barré syndrome, acute disseminated encephalomyelitis, and peripheral neuropathies [6,14,23-25]. The mechanisms underlying EBV CNS involvement are not fully understood, but proposed pathways include direct viral invasion, infection of neurons, glial cells, or endothelial cells of the blood-brain barrier, inflammatory injury, and immune-mediated mechanisms such as molecular mimicry [14,24,25].

EBV meningitis usually presents with headache, fever, and meningeal irritation, and CSF analysis typically demonstrates lymphocytic pleocytosis, elevated protein, usually preserved glucose, and occasionally atypical lymphocytes [16,22,23,25]. In our patient, the combination of sore throat, gastrointestinal symptoms, vertical nystagmus, gait instability, lymphocytic CSF pleocytosis, elevated CSF protein, positive EBV serology, and EBV DNA detection in CSF supported EBV-associated meningitis. Similar to the cases reported by Wang et al. [13] and Srifuengfung et al. [16], our case occurred in an immunocompetent young adult, showed lymphocytic CSF pleocytosis with elevated protein, had positive CSF EBV PCR testing, and resulted in a favorable outcome. However, our patient had atypical manifestations, including dizziness, gait instability, vertical nystagmus, sore throat, and gastrointestinal symptoms, while neuroimaging remained normal and no evidence of encephalitis was identified. By comparison, Wang et al. described a more classic meningitic presentation [13], whereas Srifuengfung et al. reported more severe CNS involvement with altered mental status [16]. These cases highlight the importance of considering EBV in the differential diagnosis of aseptic meningitis in immunocompetent individuals, particularly when CSF analysis demonstrates lymphocytic pleocytosis and routine bacterial investigations are negative. Nevertheless, EBV DNA detection in CSF should be interpreted cautiously because it may reflect true CNS infection, reactivation, coinfection, inflammatory CNS disease, or lymphoproliferative disorders; therefore, low-level EBV PCR positivity alone does not always prove causality [15,22-25]. The role of antiretroviral therapy in EBV-associated meningitis remains uncertain. Unlike VZV, for which IV acyclovir is considered standard therapy in CNS infection, no randomized clinical benefits of acyclovir for EBV meningitis. Consequently, current management of uncomplicated EBV meningitis is largely supportive, and antiviral therapy is generally reserved for severe CNS disease [25]. In our patient, acyclovir was initiated empirically before CSF PCR results became available because herpes simplex virus and VZV meningitis could not initially be excluded. Although the patient recovered completely, the contribution of acyclovir to this favorable outcome cannot be established, as spontaneous recovery is common in immunocompetent patients with EBV meningitis.

Compared with VZV meningitis, EBV meningitis appears to have a less distinctive prodromal phase. Several reported EBV meningitis cases presented with nonspecific constitutional symptoms, such as fever, headache, malaise, fatigue, sore throat, or symptoms resembling infectious mononucleosis [13,16], while some patients developed meningitis without any preceding systemic manifestations [13,16]. In contrast, VZV meningitis is often preceded by characteristic dermatomal pain, paresthesia, otalgia, or vesicular rash, although meningitis may also occur in the absence of cutaneous lesions. Notably, delayed appearance of the rash after the onset of neurological symptoms has been documented in immunocompetent patients with VZV meningitis, including cases in which vesicular lesions appeared approximately two days after headache and fever began, a pattern similar to that observed in the present patient [8-10,20,21]. Regarding clinical severity, both EBV and VZV meningitis most commonly present with headache, fever, photophobia, nausea, vomiting, and nuchal rigidity. However, neurological complications appear to be reported more frequently in EBV infection. Published EBV cases have described altered mental status, encephalopathy, cranial nerve involvement, seizures, and progression to meningoencephalitis [13,14,16]. In contrast, immunocompetent patients with VZV meningitis generally exhibit a more benign meningitic syndrome characterized predominantly by severe headache and fever, with preserved consciousness and normal neurological examination findings. Although VZV can cause encephalitis and vasculopathy, these manifestations are uncommon in uncomplicated meningitis cases [3,9,10]. Therefore, EBV meningitis may demonstrate a broader clinical spectrum and potentially greater neurological severity, whereas VZV meningitis more often remains confined to meningeal inflammation.

**Figure 2:**
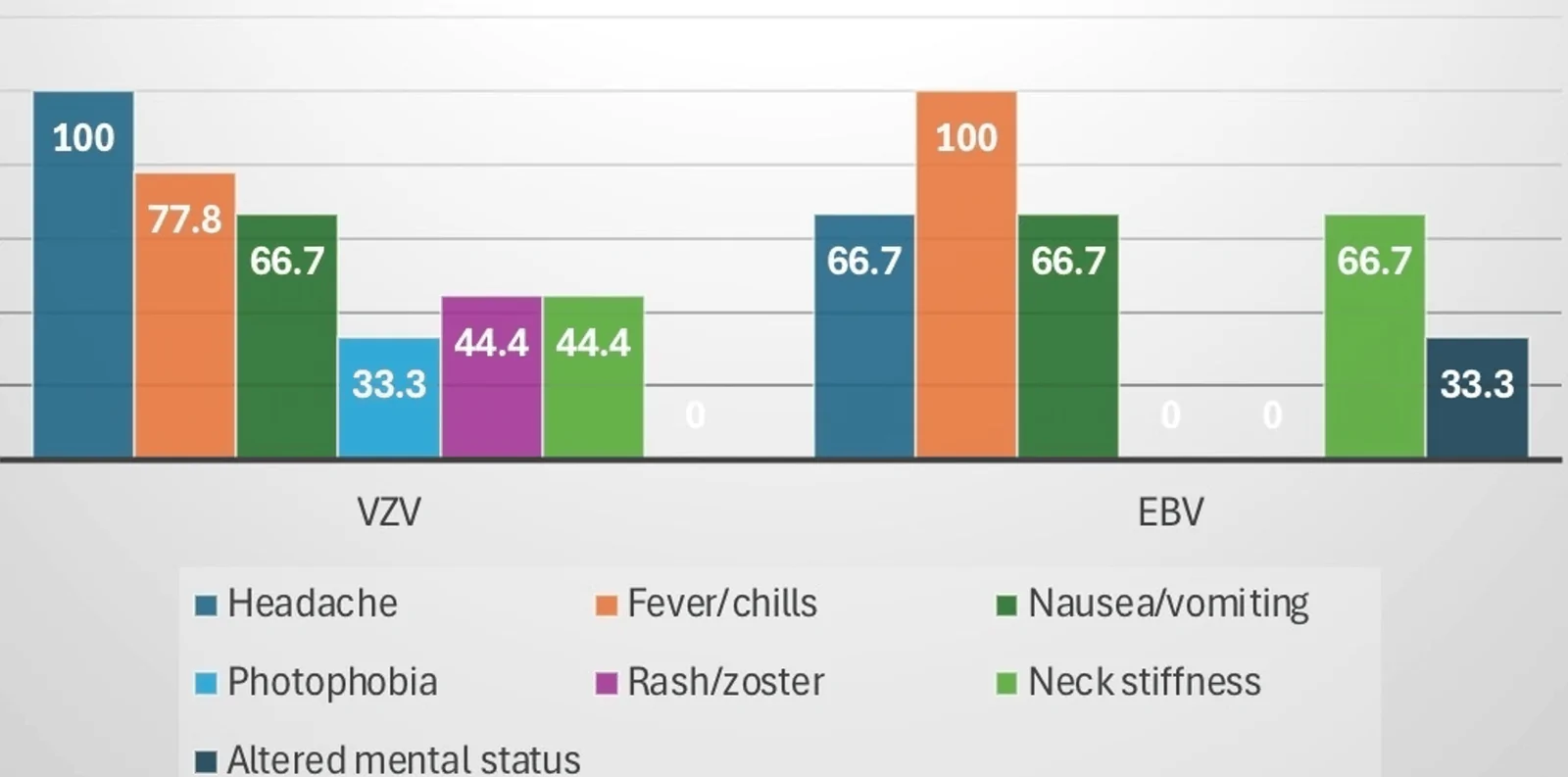
Clinical features among traceable VZV and EBV meningitis/CNS infection cases included in the present review Headache and fever/chills were common in both groups. Rash/zoster was reported only among VZV cases, whereas altered mental status was reported only among EBV-associated CNS infection cases in the reviewed data. EBV: Epstein-Barr virus; VZV: Varicella-zoster virus Figure generated by the authors based on data extracted from the reviewed literature. Source: Present cases and references [8-12,15,16].

To better visualize these clinical differences, the frequency of key presenting features among the present and previously reported VZV and EBV cases is summarized in Figure 2.

Lumbar puncture findings are broadly similar in both infections, with lymphocytic pleocytosis and elevated protein concentration being the predominant abnormalities. Nevertheless, VZV meningitis frequently demonstrates marked CSF pleocytosis and substantially elevated protein levels. In the VZV case series by Chen et al., CSF white blood cell counts ranged from 147 to 478 cells/μL, with a median protein concentration of 1.41 g/L, while glucose levels remained largely preserved [8]. Similar findings have been reported in other VZV meningitis studies [9-12,20,21]. EBV meningitis likewise demonstrates lymphocytic pleocytosis and elevated protein levels, although reported cell counts tend to be more variable and occasionally lower than those observed in VZV meningitis [13,16,22]. Glucose levels are usually normal in both conditions. Both EBV and VZV meningitis may be associated with elevated cerebrospinal fluid opening pressure, reflecting increased intracranial pressure secondary to meningeal inflammation [8,9,13,16]. Therefore, CSF findings alone may not reliably distinguish EBV from VZV meningitis, making molecular testing by PCR essential for definitive diagnosis.

**Figure 3:**
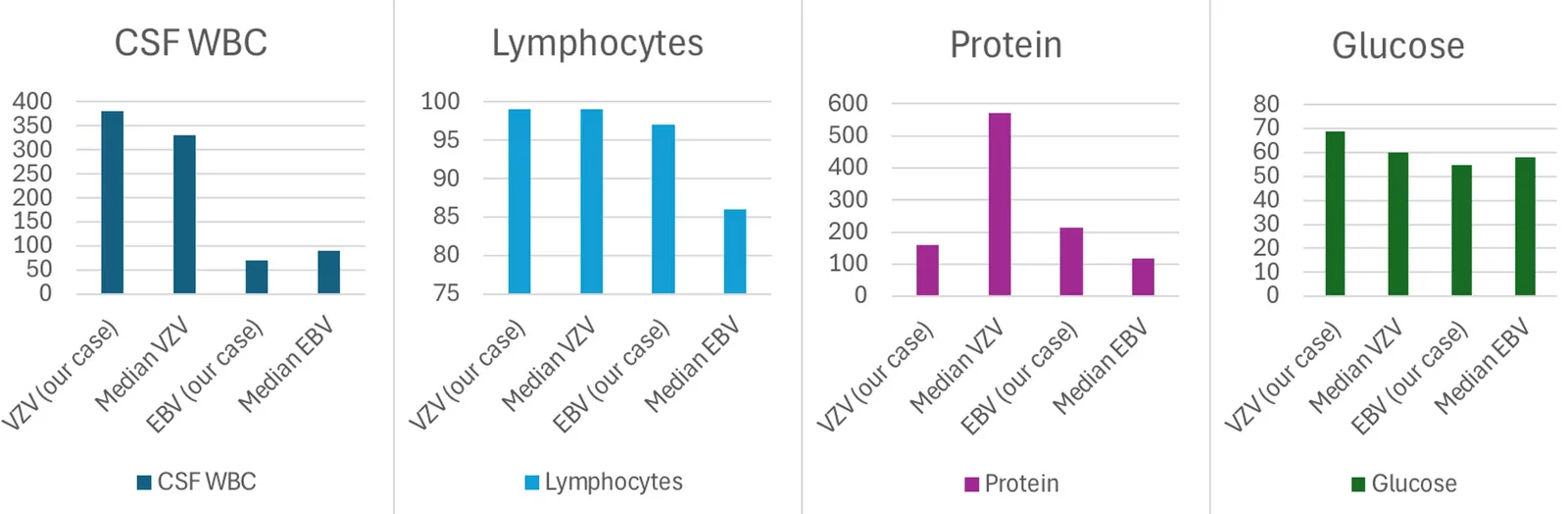
Cerebrospinal fluid comparison between the present cases and reviewed literature EBV: Epstein-Barr virus; VZV: Varicella-zoster virus This figure was generated by the authors from the extracted study data and does not reproduce any previously published figure. Source: Present cases and references [8-12,13,16]

These overlapping CSF patterns are further illustrated in Figure 3, which compares the present VZV and EBV cases with the median CSF findings reported in the reviewed literature.

The prognosis of both EBV and VZV meningitis is generally favorable in immunocompetent individuals. Most patients recover completely without permanent neurological deficits. For VZV CNS infection, intravenous acyclovir is generally recommended, whereas the benefit of antiviral therapy in EBV meningitis remains uncertain. However, severe neurological sequelae, prolonged recovery, and progression to encephalitis have been reported more frequently in EBV-associated CNS disease [14,24]. Although both VZV and EBV meningitis were associated with favorable outcomes, clinical improvement appeared more rapid in VZV cases, whereas EBV-associated disease showed a slower or more variable recovery pattern.

Overall, comparison of the present cases with previously reported cases suggests that VZV and EBV meningitis share substantial overlap in routine CSF findings, including lymphocytic pleocytosis, elevated protein, and normal or near-normal glucose levels; therefore, CSF parameters alone cannot reliably distinguish between the two infections [4,5,18,20-25]. Clinical clues may help guide suspicion: delayed, subtle, or absent vesicular rash may occur in VZV meningitis, whereas pharyngitis, lymphocytosis, transaminitis, gastrointestinal symptoms, and broader neurological manifestations may support EBV-associated disease [8-16,20-25]. In the reported cases reviewed, EBV-associated meningitis or meningoencephalitis appeared to show a more variable clinical course, with neurological findings such as dizziness, gait instability, altered mental status, or encephalitic features in some patients, while isolated VZV meningitis in immunocompetent individuals often showed rapid improvement after antiviral therapy [9,16,19-25]. However, this observation should be interpreted cautiously because the available evidence is mainly based on case reports and small series. Final diagnosis should integrate CSF PCR results, EBV serology when relevant, systemic findings, neuroimaging, exclusion of alternative etiologies, and clinical evolution.

## Conclusions

VZV and EBV should be considered in the differential diagnosis of aseptic meningitis, even in immunocompetent individuals. Although both infections share similar CSF profiles characterized by lymphocytic pleocytosis, elevated protein levels, and generally normal glucose concentrations, important clinical differences may exist. VZV meningitis is often associated with severe headache and may occur before the appearance of a characteristic vesicular rash, whereas EBV meningitis may present with more nonspecific systemic manifestations and a broader spectrum of neurological involvement. Molecular diagnosis using CSF PCR remains essential for definitive identification because routine clinical findings and CSF analysis alone are insufficient to reliably distinguish between the two infections. Early recognition and appropriate management are associated with favorable outcomes in most immunocompetent patients.
